# A graph neural network approach for hierarchical mapping of breast cancer protein communities

**DOI:** 10.1186/s12859-024-06015-x

**Published:** 2025-01-21

**Authors:** Xiao Zhang, Qian Liu

**Affiliations:** 1 Department of Applied Computer Science, University of Winnipeg, Winnipeg, MB R3B 2E9 Canada; 2 Department of Biochemistry and Medical Genetics, University of Manitoba, Winnipeg, MB R3E 0W2 Canada

**Keywords:** Protein communities, Hierarchical clustering, Graph neural network, Group LASSO, Breast cancer, Biomarker

## Abstract

**Background:**

Comprehensively mapping the hierarchical structure of breast cancer protein communities and identifying potential biomarkers from them is a promising way for breast cancer research. Existing approaches are subjective and fail to take information from protein sequences into consideration. Deep learning can automatically learn features from protein sequences and protein–protein interactions for hierarchical clustering.

**Results:**

Using a large amount of publicly available proteomics data, we created a hierarchical tree for breast cancer protein communities using a novel hierarchical graph neural network, with the supervision of gene ontology terms and assistance of a pre-trained deep contextual language model. Then, a group-lasso algorithm was applied to identify protein communities that are under both mutation burden and survival burden, undergo significant alterations when targeted by specific drug molecules, and show cancer-dependent perturbations. The resulting hierarchical map of protein communities shows how gene-level mutations and survival information converge on protein communities at different scales. Internal validity of the model was established through the convergence on BRCA2 as a breast cancer hotspot. Further overlaps with breast cancer cell dependencies revealed SUPT6H and RAD21, along with their respective protein systems, HOST:37 and HOST:861, as potential biomarkers. Using gene-level perturbation data of the HOST:37 and HOST:861 gene sets, three FDA-approved drugs with high therapeutic value were selected as potential treatments to be further evaluated. These drugs include mercaptopurine, pioglitazone, and colchicine.

**Conclusion:**

The proposed graph neural network approach to analyzing breast cancer protein communities in a hierarchical structure provides a novel perspective on breast cancer prognosis and treatment. By targeting entire gene sets, we were able to evaluate the prognostic and therapeutic value of genes (or gene sets) at different levels, from gene-level to system-level biology. Cancer-specific gene dependencies provide additional context for pinpointing cancer-related systems and drug-induced alterations can highlight potential therapeutic targets. These identified protein communities, in conjunction with other protein communities under strong mutation and survival burdens, can potentially be used as clinical biomarkers for breast cancer.

**Supplementary Information:**

The online version contains supplementary material available at 10.1186/s12859-024-06015-x.

## Background

Breast cancer (BC) is the most diagnosed cancer and one of the leading causes of cancer deaths for women worldwide [1], prompting an urgent need for innovative approaches to understand its complex biological underpinnings. The hallmarks of cancer require cancer cells to ultimately sustain uncontrollable proliferation [2], which is achieved differently in various subtypes of BC. This heterogeneity in BC requires different approaches towards prognosis and treatment for different subtypes of BC. Patients often suffer from poor prognosis of BC until metastasis has occurred, when negative effects accumulated in other bodily functions. While treatments for localized BC often result in high survival rates, metastasis can prove to be fatal [1]. The advent of high-throughput sequencing technologies has opened a new era of cancer research, enabling the detailed examination of the genetic and proteomic landscapes of tumors. However, the vast amount of data generated by these technologies poses a significant challenge, requiring sophisticated computational techniques to extract meaningful insights. Genome-wide survival models using multiple types of omics data, such as gene expression, copy number variation (CNV), and DNA methylation, have identified the prognostic relevance of each gene in the human genome [3]. Cross-omics meta-analysis could cumulate each gene’s prognostic effects at different omics levels, but these meta-prognostic significances may still be small when viewed with a single-gene focus. Likewise, individual low-frequency mutations are likely perceived to be of minor significance when compared to key driver genes that are frequently mutated in the BC population [4]. Ultimately, this isolated analysis of genes does not accurately reflect a global view of the system-level biology of BC [5].

Organizing the gene-level survival risks or mutations into gene sets has been very useful in identifying higher-order systems of genes under high survival or mutation burdens, many of which would otherwise be missed [4]. To achieve this goal, an accurate and comprehensive knowledge map of biological systems needs to be generated. These biological networks are valuable for understanding the heterogeneity of BC from a systematic view [6] and provide the ability to identify survival/mutation burdens on various biological systems. Protein–protein interaction (PPI) networks are a type of commonly used biological network in BC research [4] and can be modeled using an affinity graph, where nodes represent proteins with their features, and edges are undirected and weighted connections between the protein interactions. To better incorporate the existing knowledge of proteins into the PPI network, their amino acid sequences can be fed into the graph as node features to create a comprehensive knowledge map of biological systems. The biological systems of BC can occupy a range of biophysical scales, from individual proteins to large groups of proteins that make up functional systems. By analyzing the survival/mutation burdens at multiple scales, it is possible to detect survival/mutation burdens that would have otherwise been missed. As such, Zheng et al*.* integrated a large amount of existing protein–protein association data with their own generated PPI data of BC and constructed a hierarchical protein systems map [4]. Subsequently, they developed a group-lasso-based statistical model and successfully identified 395 systems under mutation burdens on all scales of the hierarchy which can be used as clinical biomarkers [4].

A potential limitation of the work by Zheng et al*.* is that the mutations may not have direct clinical associations. Instead, the gene-level survival risks estimated by Smith and Sheltzer in their genome-wide survival analyses [3] are more closely related to patients’ prognosis, thus using this survival information to select biological systems in the hierarchical map may identify new biomarkers. Another potential limitation of the work by Zheng et al. is that they used an unsupervised learning method called CliXO to construct the hierarchy [4]. CliXO takes the weighted graph as input, calculates the maximal cliques, and adds the identified maximal cliques to the ontology as the threshold of edge weight of this graph is lowered [7]. Although the CliXO algorithm is widely used in detecting hierarchical communities of a network [4, 8], it only considers the edge weights and requires manual tuning of several important hyperparameters that control the threshold decreasing strategies to achieve an appropriate decision. Further improvements can be made by incorporating existing knowledge of gene products and functions into the PPI network through use of terms in the Gene Ontology (GO) database [9, 10]. The three branches of the GO database—Biological Process (BP), Cell Component (CC), and Molecular Function (MF)—can bring valuable information to the PPI networks, thus allowing systematic discovery of novel hierarchical functional modules or protein communities of BC under the guidance of the existing domain knowledge.

Recently, graph neural networks (GNNs) have shown great capability in automatically analyzing biological networks as well as power in combining auxiliary data sources, like protein sequences, to achieve best model performance [11–13]. GNNs are a type of deep learning algorithm that could take node features, edge features, and graph structure into consideration for representation learning. The node features can be initialized using the protein sequence information, which will be aggregated by the features of the neighbor nodes in each layer of the GNN, and eventually arrive at the final target. Yang et al*.* proposed a GNN autoencoder to learn node representations for PPI prediction and achieved very high accuracy [14]. Likewise, Long et al*.* used the same strategy of GNN autoencoder to learn node embeddings and applied the learned embeddings on drug–target interaction (DTI) and synthetic lethality (SL) predictions [12]. In our case, the learned features could be extracted from protein amino acid sequences and passed on to several GO term supervised classification GNN modules and aggregation modules to construct the hierarchical tree which will clearly show the architecture of the biological systems and their relationship for us to understand BC heterogeneity. After the hierarchy is built, the group-lasso-based statistical method [4] can be used to identify which biological systems are under meta-survival/mutation pressure and undergo significant alterations when targeted by specific drug molecules, as well as cancer-dependent perturbations.

There are four aims for this study: 1) Use a pretrained deep learning model to extract features from protein amino acid sequence data; 2) Combine Zheng et al.’s data [4] and the features generated in the first aim to generate an affinity graph; 3) Use GO terms as guidance to construct a hierarchical tree of protein communities/biological systems for BC using GNNs; 4) Use HiSig method to pinpoint BC protein communities/biological systems that are clinical hotspots. This study could decipher the BC risks that arise from multiple unsuspecting tumor genomes and gradually converge on their higher-order entities (*e.g.* transcriptomes and proteomes). The identified protein communities under high prognostic risk, mutation pressure, drug alterations, and cancer-dependent perturbations could be potential biomarkers for BC.

## Methods

**Fig. 1:**
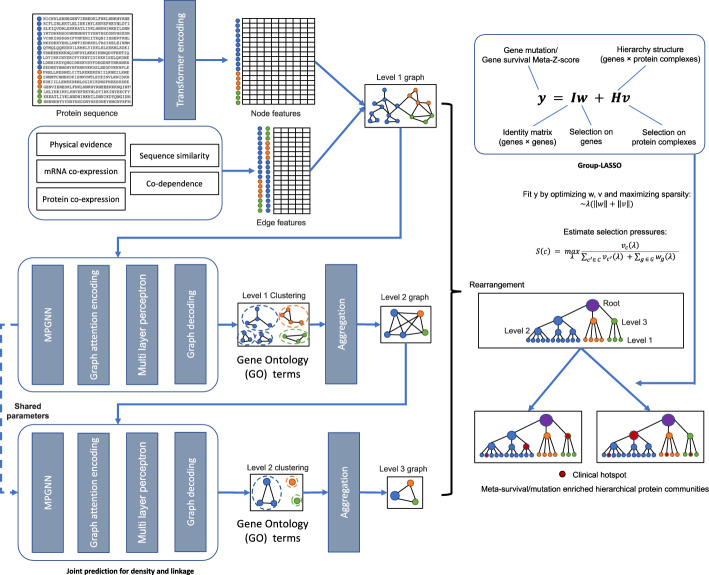
The hierarchical biological system detection framework. Protein sequence data is assembled into nodes and edges containing physical evidence, mRNA co-expression, protein co-expression, sequence similarity, and co-dependence data. The resulting graph was clustered and rearranged to produce a hierarchical tree of protein systems. Group-lasso was applied to identify clinical hotspots for mutation/survival pressure, CMap signals, and DepMap signals

An overall workflow of the proposed BC hierarchical biological system detection framework is shown in Fig. 1. A pre-trained deep learning model was used to extract 1,280 numeric features from the amino acid sequence of each protein. These numeric features were stored in the nodes of the graph (each node represents a protein). Pairwise protein association and interaction information were collected from previously published evidence and stored in the edges that link two proteins [4]. Edge information included physical interaction evidence, mRNA co-expression, protein co-expression, sequence similarity, and co-dependence. The resulting graph was input into a message passing GNN (MPGNN) block to embed edge features and node features into new node representations. The newly learned node features were then input into a supervised GNN module to predict the node GO annotations (labels). There were several GNN modules, each configured with an aggregation module to aggregate a cluster of nodes identified in current graph level into a new node in the next graph level. The new graph was input into another GNN module to repeat the clustering process. Eventually a hierarchical tree of these predicted clusters was constructed by rearranging the nodes in each graph level. All components of our methods, including layer details, model configurations, and training procedures, are available in our GitHub repository at https://github.com/maomao853/BC-Multi-Omics-GNN.

### Data sources

Seven major data sources were used in this study, including protein sequence data, PPI data, gene-level mutations, GO terms, gene-level meta-survival effects, drug-induced gene-level perturbations (CMap), and gene dependencies of BC cell lines (DepMap). The protein sequence data of 19,035 proteins was downloaded from the Uniport database, which is the world’s leading high-quality, comprehensive, and freely accessible resource of protein sequences [15]. The PPI and gene-level mutation data were downloaded from Zheng et al.’s paper. The PPI data consists of 1.8 × 10^8^ protein pairs, each with 5 scores representing association strengths of physical evidence, mRNA co-expression, protein co-expression, sequence similarity, and co-dependence. The human GO terms were downloaded from GO database [9]. There are 5,764 unique genes with annotated GO terms. The gene-level meta-survival effects were downloaded from Smith and Sheltzer’s paper [3]. They constructed a total of 3 million Cox regression models for each gene across different omics data in The Cancer Genome Atlas (TCGA) and found more than 112,000 gene features with significant survival associations. We downloaded the z-scores of these Cox models. Each gene can have 4 different z-scores corresponding to 4 omics data used in the Cox regressions: CNA z-score, gene expression z-score, DNA methylation z-score, and mutation z-score. After matching these data sources, we got the final graph with 4,968 nodes/genes/proteins, and 710,751 edges/protein pairs. Drug-induced genetic perturbation data and BC cell line gene dependency data were downloaded from the Connectivity Map (CMap) and Dependency Map (DepMap) of the Broad Institute, respectively [16, 17].

Protein data was randomly split into three datasets: train, validation, and test sets, with a ratio of 70:10:20. The large sample size of proteins used (4,968 proteins) ensured independence of the train, validation, and test datasets, with very rare homologies between them. In each iteration, the training set was used to train the GNN, while the validation set evaluated performance of hyperparameters. After many combinations of hyperparameters were tested, the best-performing model was evaluated on the test set to produce the final performance metrics. Please note that we did not implement k-fold cross-validation, as our primary objective was to incorporate GO terms for information fusion rather than to optimize model performance.

### Protein feature extraction

We utilized a recently published pre-trained deep learning model, Evolutionary Scale Modeling (ESM)-1b Transformer [18], to extract 1,280 numeric features for each protein in our dataset. ESM-1b is a 33 layers’ Transformer-based deep contextual language model with approximate 650 million parameters, which was trained on 250 million protein sequences. Please note that there are numerous ESM variants with varying layers and weights, resulting in different feature dimensions (Table S1). For example, the simplest ESM-2 model has only 8 million parameters, but its performance is significantly worse than that of more complex models. We selected ESM-1b for its optimal balance between model complexity and performance, offering rich representations without imposing excessive computational demands. It has been proven to learn deep representations from proteins with very good quality [18, 19]. The protein features generated in the first step were then used to construct an affinity graph together with the PPI scores from Zheng et al.’s study.

### Hierarchical biological system tree construction

GNN-based hierarchical clustering models, such as Hi-Lander, have been proposed to solve supervised image clustering problem and achieved state-of-art performance [20]. However, the Hi-Lander model uses *k* nearest neighbors (KNN) to initial the links/edges of the graph in different scales in the hierarchy. It cannot take the PPI into consideration, which is the key information for studying biological systems. To address this problem, we modified the initialization procedure of the first-level graph of the Hi-Lander and added an MPGNN [21] head for learning node representations from both node features and edge features. The proposed method was named MPGNN-HiLander.

The generated graph ${G}_{0}=\{V, E, {X}_{V}, {X}_{E}\}$ consisted of both node features ${X}_{V}$ (1,280 ESM-1b extracted protein features) for 4,968 nodes (*V*) and edge features ${X}_{E}$ (5 types of protein–protein association scores) for 710,751 edges (*E*). It was used as the first level graph in our hierarchical clustering framework (Fig. 1). A clustering function $\phi$ can take any graph $G$ as input and output an edge subset ${E}{\prime}\subset E$ i.e.,${E}{\prime}=\phi \left(G\right).$ The resulting graph ${G}{\prime}=\left\{V,{E}{\prime}, {X}_{V}{\prime}\right\}$(edge features ${X}_{E}$ were embedded into node features${X}_{V}$, thus are not involved in the second level and above) is then split into connected components, with each corresponding to a cluster of nodes. This single-level clustering can be generalized to multiple levels and form the hierarchy eventually. Given a graph$G=\{V, E, {X}_{V}\}$, a sequence of graphs ${G}_{l}=\{{V}_{l},{E}_{l} ,{X}_{{v}_{l}}\}$ can be generated iteratively, where $i=1\dots |{V}_{i}|$ and$l=1\dots$, were iteratively generated using a base cluster function $\phi$ and an aggregation function$\psi$. A detailed description of the aggregation function $\psi$ used in this study is provided in the supplementary materials and can also be found in the original Hi-Lander paper [20]. We constructed and realized the clustering and aggregation of second level and beyond using the default loss function used by Hi-Lander model [20].

The model’s performance on the test set was evaluated using three metrics: Pairwise F-score (Fp), BCubed F-score (Fb), and Normalized Mutual Information (NMI). The formulas for calculating these metrics are provided in the supplementary materials (Equation S3, S6, S7). CliXO [4], GCN-V [22], and GCN-V + E [22] were used as baselines. After the model was trained, we applied it to the whole dataset to generate a hierarchical map of the biological system for downstream analysis.

### Clinical hotspot detection

After the hierarchical tree was built, the HiSig algorithm (Eq. 1) [20] was applied to detect which biological systems were under mutation/survival pressure. Adapting the HiSig algorithm (Eq. 1) [20] to work on CMap and DepMap datasets enabled it to highlight biological systems which contain genes dependencies of BC cell lines and corresponding drugs that cause gene-level perturbations.

1 $$y = Iw + Hv$$

Here, *y* is the corrected mutation counts or meta-z-score (${z}_{meta}$) for all genes, *I* is an identity matrix with the size of genes by genes. *H* is a gene by systems matrix—if a gene is a member of a biological system, the corresponding element will be 1, otherwise it will be 0. While *w* and *v* are weight matrices to be estimated. ${z}_{meta}$ was calculated using Eq. 2.

2 $${z}_{meta} = \frac{{\sum }_{i=1}^{K}{Z}_{i}}{\sqrt{K}}$$

where K is the number of platforms, in this case, K = 4 (CNV, gene expression, DNA methylation, mutation). And the ${Z}_{i}$ are calculated by dividing the coefficient $\beta$ in the Cox regression model (Eq. 3) by its standard error. The univariate Cox regression model used to generate the z-scores could be formulated as follows:

3 $$h(t,X) = {h}_{0}(t){e}^{\beta X}$$

where *t* is the survival time, $h(t,X)$ is the hazard function, ${h}_{0}$(t) is the baseline time-dependent risk of death, $X$ is a time-independent covariate matrix for a set of genes. It can be gene-level CNV, gene expression, DNA methylation, and mutation.

By solving Eq. 1 in a LASSO regulated way—fit y by optimizing w, v and maximizing sparsity:$\sim \lambda (\Vert w\Vert +\Vert v\Vert )$—the weight vectors *w* and *v* could be estimated under different $\lambda$. They are modeling the positive mutation/survival selection pressures on genes and systems, respectively. We tested 500 different $\lambda$ values, which yielded 500 optimal solutions of *w* and *v*. Each biological system c was then assigned a selective pressure *S(c)* over all $\lambda$ values. The selective pressure *S(c)* is defined as the maximum fraction of the weight of a biological system *c* among all weights:

4 $$S\left( c \right) = \mathop {max}\limits_{\lambda } \frac{{v_{c} \left( \lambda \right)}}{{\sum _{{c^{\prime} \in C}} v_{{c^{\prime}}} \left( \lambda \right) + \sum _{{g \in G}} w_{g} \left( \lambda \right)}}$$

The statistical significance of the *S(c)* was tested in a permutation way. First, an empirical *P *value was calculated by comparing *S(c)* of the actual hierarchy against 10,000 random hierarchies in which the hierarchy structure H is permuted with respect to gene labels (i.e., permuting the rows in *H*). Then the false discovery rate (FDR) was calculated using the Benjamini–Hochberg procedure, and a pre-defined cut-off of FDR < 0.25 was used to select the significant biological systems (clinical hotspots).

### Hierarchical biological system tree annotation

Visualization and annotation of the hierarchical biological system tree was performed using Cytoscape [23]. Due to the large number of nodes and insignificance of certain nodes, the tree was pruned to provide useful information. Pruning was conducted manually using a bottom-up breadth first search (BFS) method of traversal, where any insignificant non-leaf nodes were removed from the tree (insignificant nodes are defined as any node that is not a clinical hotspot). An approach using bottom-up BFS allows us to visit most non-inclusive nodes first since BFS explores all nodes at the present depth before moving on to the next depth. This ensures we do not miss any insignificant nodes that need pruning and avoid pruning significant nodes.

The annotations built upon enrichment analysis of gene sets at various levels, from individual genes to biological systems, using Enrichr [24] and the Kyoto Encyclopedia of Genes and Genomes (KEGG) 2021 Human gene-set library [25]. Enriched terms were evaluated using q-value, odds ratio, and a combined score for the term in the gene-set context. Significant overlapping terms and contextual information were used to determine the main function of gene sets from individual to system-level and used to annotate biological systems at all levels. Naming of the biological systems were performed in-house using biological knowledge and literature analysis. The naming process only serves to outline system functions at a high-level and does not affect analysis of the hierarchical tree.

### Evaluation of identified clinical hotspots

Since the biological systems were identified from BC cell line data and publicly available cancer PPI information, we were able to further evaluate their significance in BC cohorts. Alterations of biological systems with survival and mutation significance in HiSig analysis were checked in the METABRIC cohort using the CBioPortal OncoPrint function [26]. The survival difference between the altered patients and unaltered patients were also evaluated for each significant biological system.

## Results

The performance of the proposed MPGNN-HiLander and baselines can be found in Table 1. The proposed MPGNN-HiLander achieved significantly better performance than baseline models of CliXo, GCN-V, and GCN-V + E across all metrics.

**Table 1:** Annotation prediction performance using various branches of data from the GO database. GO branches used includes biological process, cell component, molecular function, and gene ontology. Performance was measured using Pairwise F-score (Fp), BCubed F-score (Fb), and Normalized Mutual Information (NMI). The CliXo, GCN-V, and GCN-V + E models served as baseline measurements and the proposed model, MPGNN-HiLander, resulted in the highest accuracy across all metrics and datasets

Method	Biological process			Cell component			Molecular function			Gene ontology		
	Fp	Fb	NMI	Fp	Fb	NMI	Fp	Fb	NMI	Fp	Fb	NMI
CliXO	0.08	0.15	0.51	0.05	0.09	0.49	0.06	0.13	0.59	0.11	0.13	0.57
GCN-V	0.24	0.31	0.66	0.27	0.29	0.69	0.24	0.35	0.60	0.23	0.26	0.59
GCN-V + E	0.24	0.32	0.67	0.31	0.29	0.68	0.28	0.34	0.62	0.24	0.31	0.60
MPGNN-HiLander	0.52	0.50	0.71	0.57	0.59	0.77	0.60	0.55	0.69	0.54	0.56	0.67

**Fig. 2:**
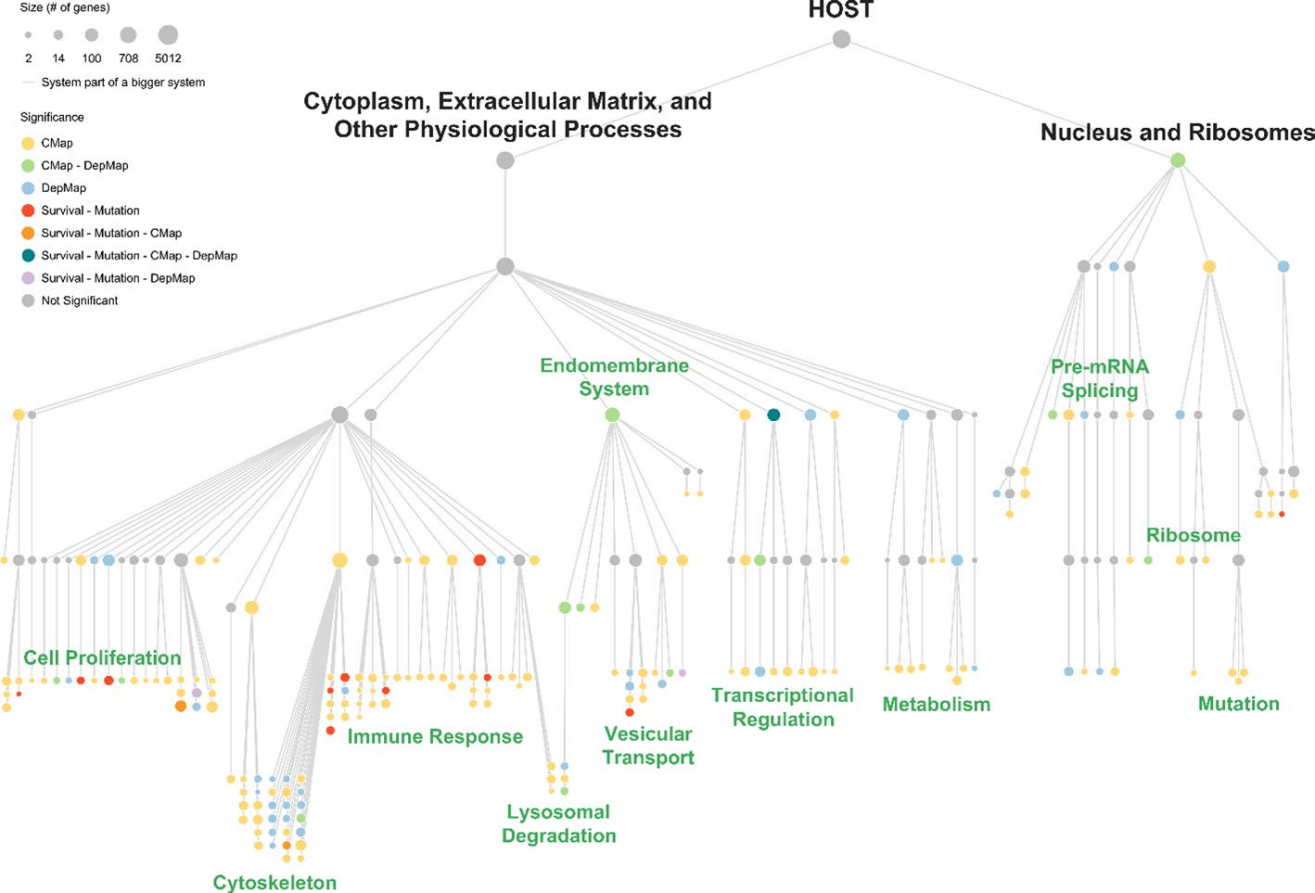
Hierarchical biological system tree (HOST) produced by the MPGNN-HiLander model. Nodes represent protein systems and edges represent low-order systems which belong to respective higher-order systems. Individual nodes are distiguished by size representing number of genes and colour representing significance in group-lasso

There were 878 biological systems identified by the MPGNN-HiLander model that formed a 6-layer hierarchy (Fig. 2) which we called HOST (Hierarchical Organization of Systems with Tumors). After the tree was pruned, 249 biological systems and 191 clinical hotspots remained. The distribution of the clinical hotspots has 28 hotspots in the right subtree (Nucleus and Ribosomes) and 163 hotspots in the left subtree (Cytoplasm, Extracellular Matrix, and Other Physiological Processes). Detailed information of the hierarchy can be found in supplementary material Data S1.

**Fig. 3:**
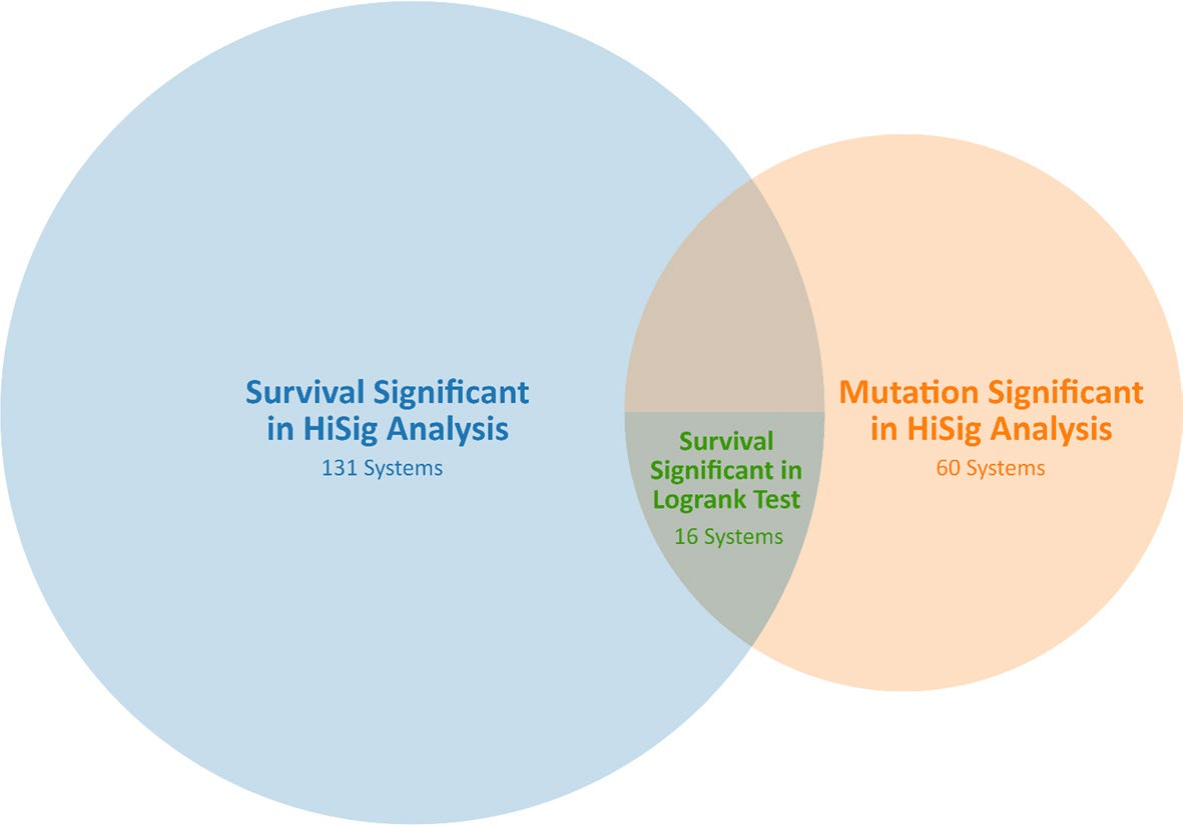
Overlap of significant protein systems. The 131 systems with significant survival pressure in HiSig analysis and 60 systems with significant mutation pressure in HiSig analysis have 16 overlapping systems in common. These 16 systems contained only 8 systems that have overlapping prognostic value with logrank tests conducted on the METABRIC cohort

Among the 878 biological systems, 131 are under significant survival pressure (Table S2) and 60 are under significant mutation pressure (Table S3). There is an overlap of 16 biological systems that are under both survival and mutation pressures (Fig. 3), of which 8 systems are significant for prognosis within the METABRIC cohort. HOST:280 was identified as one of these systems and contained the genes BRCA1 and BRCA2.

The list of 8 biological systems with significant prognostic value, determined by HiSig analysis and logrank tests on the METABRIC cohort, was further narrowed down to 3 systems by presence of BC cell dependencies. In total, there are 50 significant DepMap signals that describe gene dependencies of breast cancer cell lines (Table S4). The systems with BC cell line gene dependencies overlapped with the 8 previously identified significant biological systems on 2 significant biological systems HOST:37 and HOST:861, which fall under the systems of Transcriptional Regulation and Cell Proliferation respectively (Fig. 3). A knockout of HOST:37 genes in the T-47D cell line results in significant survival pressure. Similarly, a knockout of HOST:861 genes in the CAL-120 cell line also results in significant survival pressure. These gene dependencies of CAL-120 and T-47D cell lines are shown in Table 2 and can serve as potential biomarkers due to their accumulated significance from survival/mutation pressure, prognosis, and BC cell dependencies.

**Table 2:** Significant biological systems containing cellular dependencies of breast cancer cell lines. The significance of these biological systems was determined by survival pressure, mutation pressure, and prognostic value, with q-value < 0.05 indicating significance

Dependent system	Cell line	*p*-value	*q*-value
HOST:37	T-47D	0.002	0.0488
HOST:861	CAL-120	0.002	0.0475

**Fig. 4:**
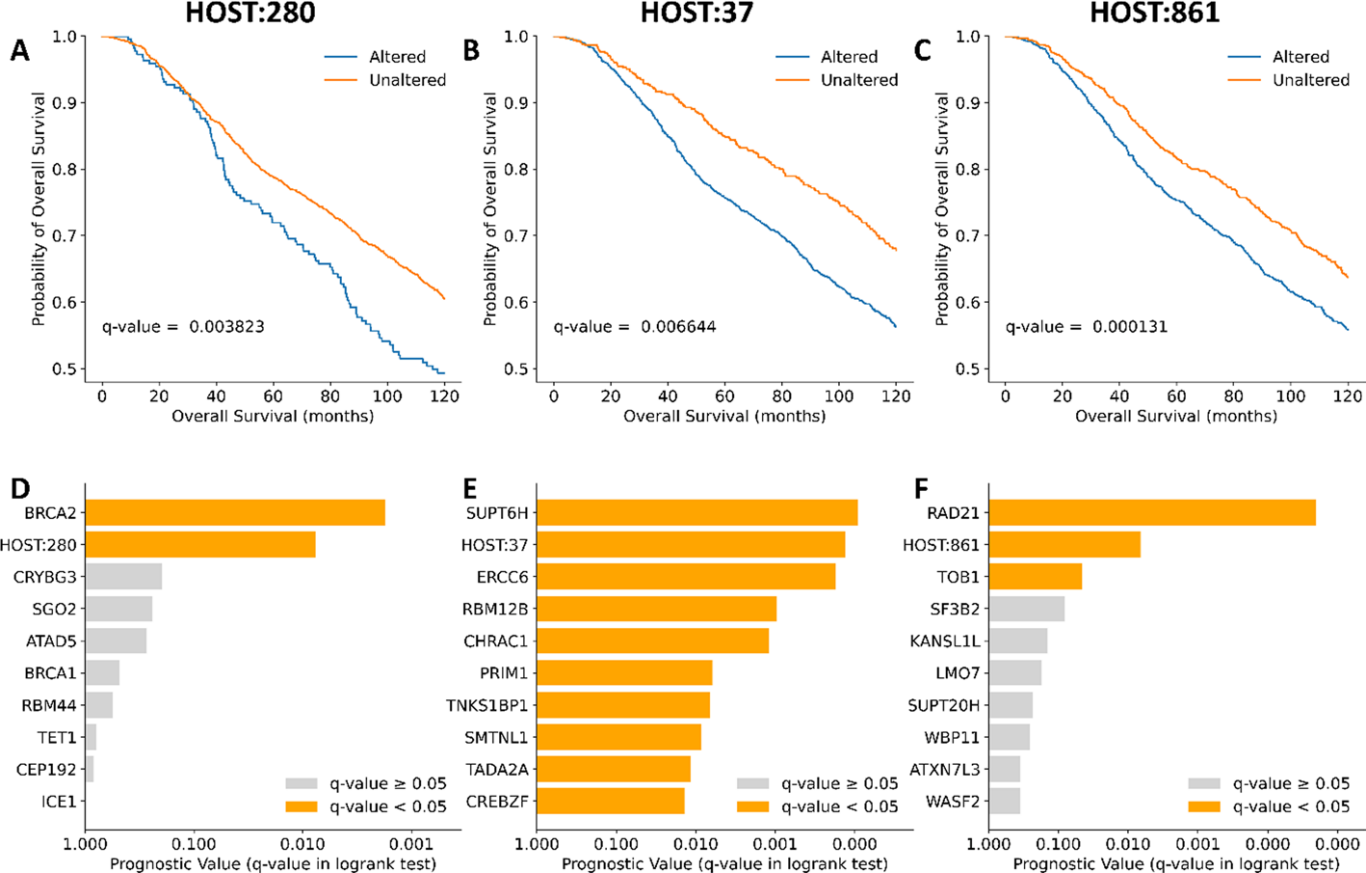
Clinical significance and prognostic value of protein systems. Kaplan–Meier curves [(**A**) to (**C**)] show survival differences between patients with altered and unaltered genes in protein systems throughout 120 days. Bar plots [(**D**) to (**F**)] show prognostic value of protein systems and their individual genes for determining survival differences of altered and unaltered protein systems. [(**A**) and (**D**)] HOST:280, Immune Response. [(**B**) and (**E**)] HOST:37, Transcriptional Regulation. [(**C**) and (**F**)] HOST:861, Cell Proliferation

**Fig. 5:**
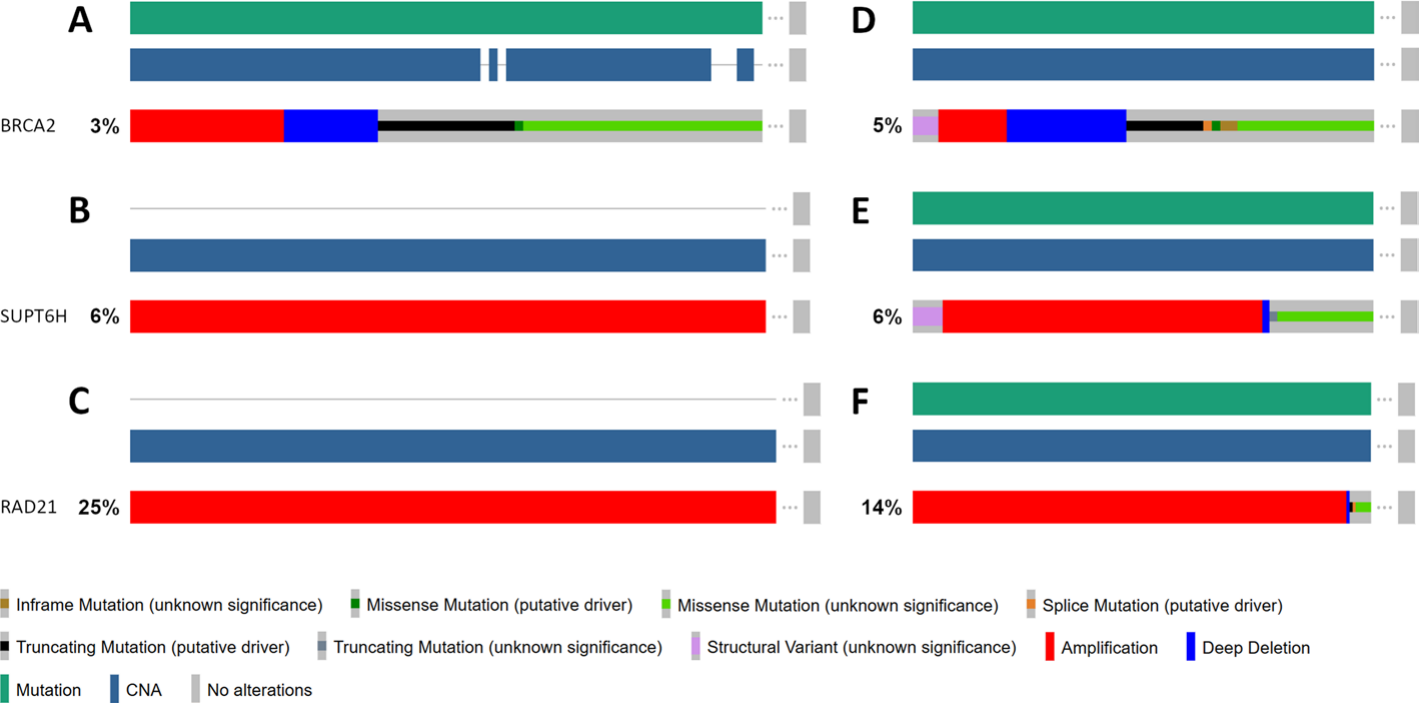
Genetic alterations of significant genes in HOST:280, HOST:37, and HOST:861. BRCA2 in system HOST:280 [(**A**) and (**D**)], SUPT6H in system HOST:37 [(**B**) and (**E**)], and RAD21 in system HOST:861 [(**C**) and (**F**)] were queried in the METABRIC dataset [(**A**) to (**C**)] and TCGA PanCancer Atlas [(**D**) to (**F**)]

BC patients with genetic alterations in these three biological systems suffered poor survival outcomes compared to patients without genetic alterations (Fig. 4). The 10-year survival differences of METABRIC patients with altered and unaltered genes in the three biological systems can be found in Fig. 4 and the genetic alterations of these systems in the METABRIC cohort are shown in Fig. 5.

All three systems HOST:280, HOST:37, and HOST:861 had significant prognostic value compared to individual genes, ranking as second most significant in a logrank test (Fig. 4D to F). Three genes—BRCA2, SUPT6H, and RAD21—had the most prognostic value in HOST:280, HOST:37, and HOST:861 respectively. The gene BRCA2 had the most putative driver mutations in METABRIC (Fig. 5A) and TCGA-BRCA (Fig. 5D) cohorts. It had the rarest frequency of occurrence of the three genes examined, with mutations in 3% (METBABRIC) and 5% (TCGA-BRCA) of patients profiled. SUPT6H and RAD21 were both more frequently mutated than BRCA2. However, these two genes contained mostly amplifications and few putative driver mutations.

Furthermore, CMap signals are seen in 144 biological systems which show significant survival differences when exposed to various small molecules. Overlap of survival pressure, mutation pressure, prognosis value, and cellular dependency converges on HOST:37, which is a high-level biological system in level 3 of HOST, encompassing 3 child systems involved in transcriptional regulation (Fig. 2). A total of 115 small molecules were identified to cause gene perturbation in HOST:37, including approved drugs, experimental drugs, and other chemical perturbagens. A total of 38 FDA approved drugs were extracted from the list, of which four are currently used for BC treatment—exemestane, fulvestrant, paclitaxel, and vinblastine. The respective dose and duration that cause the most perturbation for each drug was chosen to be shown in Table 3.

**Table 3:** FDA approved drugs that target the system HOST:37, Transcriptional Regulation. A total of 38 unique drugs were identified. Drugs with different possible doses and durations were selected based on significance in alteration of target survival (lowest *q*-value)

Small molecule	Dose (M)	Duration (h)	*p*-value	*q*-value
Alpha-estradiol	1.00E−08	6	0.002	0.0488
Alprostadil	1.00E−05	6	0.002	0.0475
Amitriptyline	1.00E−06	6	0.002	0.0408
Azathioprine	1.00E−04	6	0.002	0.0439
Celecoxib	1.00E−05	6	0.002	0.0418
Chlorpromazine	1.00E−06	6	0.002	0.0408
Chlorpropamide	1.00E−04	6	0.002	0.0488
Ciclosporin	1.00E−06	6	0.002	0.0428
Clofibrate	1.00E−04	6	0.002	0.045
Colchicine	1.00E−07	6	0.002	0.0408
Daunorubicin	1.00E−06	6	0.002	0.0428
Deferoxamine	1.00E−04	6	0.002	0.0488
Dinoprostone	1.00E−05	6	0.002	0.0428
Exemestane	1.00E−08	6	0.002	0.0462
Fludrocortisone	1.00E−06	6	0.002	0.0428
Fulvestrant	1.00E−06	6	0.002	0.0399
Haloperidol	1.00E−05	6	0.002	0.0408
Imatinib	1.00E−05	6	0.002	0.0408
Irinotecan	1.00E−04	6	0.002	0.0439
Lomustine	1.00E−04	6	0.002	0.0418
Mercaptopurine	1.00E−04	6	0.002	0.0408
Metformin	1.00E−05	6	0.002	0.045
Monorden	1.00E−07	6	0.002	0.0418
Nifedipine	1.00E−05	6	0.002	0.0428
Orlistat	1.00E−05	6	0.002	0.0475
Paclitaxel	1.00E−07	6	0.002	0.045
Pioglitazone	1.00E−05	6	0.002	0.0408
Prochlorperazine	1.00E−05	6	0.002	0.0439
Sirolimus	1.00E−07	6	0.002	0.0475
Sodium phenylbutyrate	1.00E−03	6	0.002	0.045
Sulfasalazine	1.00E−04	6	0.002	0.0475
Thalidomide	1.00E−04	6	0.002	0.0439
Thioridazine	1.00E−05	6	0.002	0.0462
Tioguanine	1.00E−05	6	0.002	0.0475
Tretinoin	1.00E−06	6	0.002	0.0399
trifluoperazine	1.00E−05	6	0.002	0.0439
Valproic acid	5.00E−04	6	0.002	0.0418
Vinblastine	1.00E−07	6	0.002	0.0475

## Discussion

### Graph Neural Network

The pipeline established for constructing a multi-level protein system visualization by Zheng et al. used the CliXo model to cluster proteins into a multi-system map. However, CliXo is limited by inherent properties of unsupervised learning models. It has significant dependence on tuning hyperparameters, which requires careful manual work and may result in lots of variability if not tuned correctly. An alternative approach used in the hierarchical protein system tree, HOST, leveraged MPGNN technology to integrate additional contextual data into the clustering process. The comparison between CliXo and MPGNN-HiLander, an unsupervised and supervised learning model respectively, in Table 1 is inherently unfair with many disadvantages towards CliXo. Thus, the numbers should not be taken as a direct comparison, but rather an alternative approach to an established technique. The proposed GNN-based hierarchical clustering model performed very well on the graph-structured BC PPI data. The tree structure clearly defined associations between protein systems and allowed easy construction of a multi-system visualization of proteins. It successfully identified hierarchically structured protein systems from protein sequence data, pair-wise protein interaction/association data, and GO annotation data, which could help us understand the heterogeneity of BC systematically.

### Breast Cancer Biomarkers

In addition, the survival and mutation pressure tests successfully pinpointed 16 statistically significant overlapping hotspots within the HOST system. Among them, the system HOST:280 contains BRCA2, which is a known driver of BC when mutated [27], and thus helped establish the validity of our model. BRCA2 is a tumor suppressor gene and codes for proteins involved in transcription, DNA repair of double-stranded breaks, and recombination. Mutations in BRCA2 are responsible for more than 40% of inherited BCs [27]. Although the mutations in BRCA2 have long been noticed to play important roles in BC, they are often missed in previous genome wide analyses because of their rare mutation frequency in population. BRCA2 is the most significant prognostic gene in HOST:280 with putative drivers caused by missense and truncating mutations but is only altered in 3% (74 out of 2509 patients profiled) in the METABRIC cohort and 5% (54 out of 1084 patients profiled) in the TCGA PanCancer Atlas cohort (Fig. 5). Despite its low mutation rate, BRCA2 can be identified as a BC hotspot from accumulated effects in the other 7 genes of HOST:280, which is exactly what the proposed hierarchical map wants to achieve. Similarly, SUPT6H was identified as a potential biomarker despite having a relatively low mutation rate of 5–6% across METABRIC and TCGA PanCancer Atlas cohorts. As expected, significant systems exhibiting high mutation rate, such as RAD21 with 22% in METABRIC and 14% in TCGA PanCancer Atlas, were also able to be identified. The systems HOST:37 and HOST:861 contained SUPT6H and RAD21, which had no previously identified putative driver mutations in either cohort. Patients in the METABRIC cohort only expressed amplifications of SUPT6H and RAD21, which can possibly serve as prognostic markers for future patients. Similarly, TCGA PanCancer Atlas showed mostly gene amplifications and a few mutations of unknown significance for SUPT6H and RAD21. These unknown mutations can be further examined and potentially identified as putative drivers which may serve as biomarkers. Further evaluation of prognostic significance in the METABRIC cohort with a logrank test (Fig. 4) resulted in 8 clinical hotspots that have the potential to be developed as BC prognostic biomarkers (HOST:37, 134, 280, 281, 397, 578, 841, 861). The 10-year survival differences (Fig. S1) showed that BC patients in METABRIC with genetic alterations in these 8 biological systems suffered poorer prognosis than patients without genetic alterations.

### Gene dependencies of breast cancer cell lines

Biomarkers in BC often focus on obvious genes in line with hallmarks of cancer [2] that contribute to malignant tumor growth, particularly oncogenes and tumor suppressor genes (i.e. BRCA1 and BRCA2). However, this limited scope hides any loosely connected genes or gene sets which could provide significant prognostic value or serve as a target for treatment. A combination of different BC cell lines can be used to simulate BC tissue in vitro. Creating gene knockouts of various BC cell lines based on genes in the identified protein systems, it is possible to determine if any genes or systems are cellular dependencies of BC cell lines. This process was used to curate the DepMap database, which allows the detection of genes influencing cancer cells at the cellular level, which may be missed at the gene level. The systems, HOST:37 and HOST:861, were identified as cellular dependencies of the T-47D and CAL-120 cell lines respectively. T-47D is a cell line derived from invasive ductal carcinoma with presence of estrogen receptors and progesterone receptors [28]. CAL-120 is derived from adenocarcinoma without presence of the three main receptors (ER, PR, and HER2) [28]. These cell lines helped to confirm the impact of HOST:37 and HOST:861 protein systems towards BC tumor development and reinforce the heterogeneity of BC. Since both cell lines have wild-type BRCA1, their gene dependencies would be missed by traditional screening and prognosis as mentioned earlier. However, searching for cellular dependencies within multi-level protein systems revealed important biomarkers for prognosis. System-wide analysis provides more significant prognostic results compared individual genes, aside from outliers, such as SUPT6H in system HOST:37 and RAD21 in system HOST:861 (Fig. 4).

The system HOST:37 has functions in cell proliferation, with genes coding for basal transcription factors, RNA polymerase, and other related functions. This contributes to one of the fundamental hallmarks of cancer, “sustaining proliferative signalling” [2]. The protein encoded by SUPT6H of HOST:37 provides the highest prognostic value in the METABRIC cohort (Fig. 4). It controls expression of estrogen receptor alpha (ER $\alpha$) through mediating transcriptional elongation as a histone chaperone [29]. A decrease in SUPT6H protein levels, possibly due to mutations, could cause an increase in tumor growth due to unwinding of chromatin and thus exposing it for unhindered expression of ER $\alpha$ [29]. Despite encoding an individual protein, SUPT6H is a strong indicator of predictive outcomes and acts as a potential therapeutic target. Moreover, large protein systems can support discovery of individual (or systemic) genetic perturbations, such as the case with SUPT6H. Similarly, the system HOST:861 contributes to “sustaining proliferative signalling” [2] through direct regulation of cell proliferation. RAD21 encodes a protein in this system that provides the most prognostic value compared to other proteins (Fig. 4). The protein complex produced by RAD21 is essential for repair of DNA double-strand breaks. It is also part of a cohesin complex which is responsible for the cohesion of sister chromatids during the cell cycle [30]. Both functions are essential for cell proliferation, therefore supporting RAD21’s prognostic value and potential as a drug target. Aside from RAD21, the system HOST:861 remains most significant for prognosis and can expand the surface area for prognosis developing therapeutic targets by considering non-essential, supporting genes in the system.

### Proposed drug treatments for breast cancer

The novel biomarkers identified by the MPGNN-HiLander model, HOST:37 and HOST:861, also have the potential as targets for BC treatment. Overlap of CMap signals and biomarker systems showed 115 unique chemical perturbagens (Table S5) of the HOST:37 system. These small molecules were screened for FDA approval, producing a list of 38 FDA-approved drugs (Table 3) which cause significant perturbation in the HOST:37 system. Within this list, 4 drugs approved for BC treatment have been identified–exemestane, fulvestrant, paclitaxel, and vinblastine. Exemestane is an androgen analogue which acts as a suicide inhibitor of enzyme aromatase [31]. This inhibits the production of upstream precursors to estrogen, particularly in postmenopausal women [31]. Although estrogen is essential in normal breast tissue development, it also contributes significantly to the proliferation of cancerous breast tissue cells in the system HOST:37. By decreasing estrogen levels, BC proliferation is also suppressed [31]. Fulvestrant slows tumor growth by decreasing estrogen levels, but is targeted for postmenopausal women with BC [32]. Paclitaxel disrupts the cell cycle by inhibiting microtubule depolymerization, which results in cell cycle arrest in the G2 phase [33]. Vinblastine also disrupts the cell cycle by targeting microtubule formation. It acts by binding to tubulin molecules at both ends, leading to microtubule catastrophe [34]. Together, these drugs have a promising potential to target the system HOST:37.

Other FDA approved drugs were evaluated based on mechanism of action and target. Drugs which target Transcription Regulation of HOST:37 were identified. These drugs can potentially be repurposed as therapeutic agents targeting BC subtypes with mutation in HOST:37 functions. Based on perturbagen impact (q-value), three most significant drugs (lowest q-value) were selected from Table 3—mercaptopurine, pioglitazone, and colchicine. Mercaptopurine is a purine antagonist prodrug [35]. By acting as a purine analog (based on hypoxanthine), it can incorporate into nucleobases [35]. These nucleobases are inactivated, thus reducing purine available for DNA, RNA, and protein synthesis. This can potentially suppress tumor growth by hindering normal cell function through transcriptional inhibition and slows down DNA synthesis in the S phase of the cell cycle. Mercaptopurine has also been used in multidrug combination therapy. The combination of 6-mercaptopurine and Chlorambucil has shown to increase bioavailability, which allowed the drugs to better disrupt the cell cycle [36]. Pioglitazone is a thiazolidinediones (TZD) which activates peroxisome proliferator-activated receptor gamma (PPAR-γ) and alters transcriptional levels of genes involved in glucose and lipid metabolism. While pioglitazone is conventionally used as an anti-diabetic drug, it may potentially have a significant impact on BC tumor cells [37]. This change in gene expression ultimately results in increased insulin sensitivity and decreased glucose levels. As the Warburg Effect demonstrated that tumor cells exhibit higher rates of glucose consumption to maintain an elevated level of cell proliferation [38], a reduction in available glucose could suppress tumor growth. Since PPAR-γ is abundant in adipose tissue and female breasts can contain 7–56% adipose tissue (by volume), pioglitazone has potential to significantly suppress BC tumor growth [39, 40]. Colchicine exhibits similar functionality as current BC treatments, paclitaxel and vinblastine, which disrupt tubulin synthesis and suppresses DNA replication in the cell cycle [41]. The lack of tubulin hinders the function of microtubule-organizing centers (MTOCs) which halts cells in metaphase of the mitotic cycle and suppresses cell proliferation. Previous studies have shown anti-tumor and anti-migration effects of colchicine on gastric cancer cells [42].

### Limitations

There are some limitations in this study. First, to match the GO annotations, we significantly decreased the number of proteins from 19,035 to 4,968 and decreased the number of pair-wised protein interactions/associations from 1.8 × 10^8^ to 710,751 in the graph. Although this makes the analysis more concise and efficient, some information may be missed. Additionally, hierarchical tree annotation and data visualizations required manual effort to easily interpret the data. However, it is difficult to further streamline the process aside from automating the pipeline between GNN output and Cytoscape network input. More biological evaluations and discussions, such as gene set enrichment analysis, may be easier when visualizations are added. A future direction is the incorporation of more experiments to evaluate drug-response information from CMap by testing them on BC cell lines and other tumor models.

## Conclusion

In conclusion, we proved that the GNN model can organize structured proteomics data into a clinically valuable hierarchy. This hierarchical system tree provides a new perspective to explore BC heterogeneity whereby multi-level screening will increase the chance of identifying the rare mutations and minor survival hazards. The biological systems HOST:37 (Transcriptional Regulation) and HOST:861 (Cell Proliferation) have been identified as potential biomarkers for prognosis with the help of accumulated effects in their gene sets. Individual genes of significance include SUPT6H and RAD21, from HOST:37 and HOST:861 respectively, which have the potential to serve similar roles as BRCA2 in BC prognosis. Additionally, drugs identified by high perturbation impact can possibly be repurposed as therapeutic agents for BC. Three FDA-approved drugs—mercaptopurine, pioglitazone, and colchicine—have been identified for this purpose. Overall, this study has promising potential to map new clinically relevant information to the identified hierarchy that provides different types of biomarkers for BC.

## Supplementary Information

Additional file 1.

Additional file 2.

Additional file 3.

Additional file 4.

Additional file 5.

Additional file 6.

Additional file 7.

## Data Availability

The protein sequence data of 19,035 proteins was downloaded from the Uniport database [15]. The Protein Protein Interaction (PPI) and gene-level mutation data were downloaded from Zheng et al.’s paper [4]. These PPI data consist of 1.8 × 10^8 protein pairs, each with 5 scores representing association strengths of physical evidence, mRNA co-expression, protein co-expression, sequence similarity, and co-dependence. The human Gene Ontology (GO) terms were downloaded from GO database [9]. We downloaded the z-scores of the Cox models from Sheltzer’s paper [3].
