# Supplementary material for: A graph neural network approach for hierarchical mapping of breast cancer protein communities
Source: BMC Bioinformatics. 2025 Jan 21;26:23. doi: 10.1186/s12859-024-06015-x (PMC11749236; doi:10.1186/s12859-024-06015-x)
Supplement: Supplementary file 2 — Additional file 2. [file 12859_2024_6015_MOESM2_ESM.docx]

# Supplementary Text

# Aggregation function $\boldsymbol{\psi}$:

To clarify the aggregation function $\psi$, let $\boldsymbol{c}_{\boldsymbol{i}}^{\mathbf{(}\boldsymbol{l}\mathbf{)}}$ to be the i-th connected component in $\boldsymbol{G}_{\boldsymbol{l}}^{\mathbf{'}}$**.** For the next graph level, $\boldsymbol{G}_{\boldsymbol{l}\mathbf{+1}}$, this component is represented as a new node $\boldsymbol{v}_{\boldsymbol{l}}^{\mathbf{(}\boldsymbol{l}\mathbf{+1)}}$. Two feature vectors are assigned to this new node: an identity feature ${\tilde{\boldsymbol{h}}}_{\boldsymbol{i}}^{\mathbf{(}\boldsymbol{l}\mathbf{+1)}}\mathbf{=}{\tilde{\boldsymbol{h}}}_{\boldsymbol{m}_{\boldsymbol{i}}}^{\mathbf{(}\boldsymbol{l}\mathbf{)}}$, where $\boldsymbol{m}_{\boldsymbol{i}}\mathbf{=}$ $\underset{\boldsymbol{j}\boldsymbol{\in}\boldsymbol{c}_{\boldsymbol{i}}^{\mathbf{(}\boldsymbol{l}\mathbf{)}}}{\mathbf{argm}\boldsymbol{ax}} {\hat{\boldsymbol{d}}}_{\boldsymbol{j}}^{\mathbf{(}\boldsymbol{l}\mathbf{)}}$, representing the peak node index in $\boldsymbol{c}_{\boldsymbol{i}}^{\mathbf{(}\boldsymbol{l}\mathbf{)}}$, and an average feature ${\bar{\boldsymbol{h}}}_{\boldsymbol{i}}^{\mathbf{(}\boldsymbol{l}\mathbf{+1)}}\mathbf{=}\frac{\mathbf{1}}{\mathbf{|}\boldsymbol{c}_{\boldsymbol{i}}^{\mathbf{(}\boldsymbol{l}\mathbf{)}}\mathbf{|}}\sum_{\boldsymbol{\in}\boldsymbol{c}_{\boldsymbol{i}}^{\mathbf{(}\boldsymbol{l}\mathbf{)}}} {\tilde{\boldsymbol{h}}}_{\boldsymbol{j}}^{\mathbf{(}\boldsymbol{l}\mathbf{)}}$, which averages the features of nodes in $\boldsymbol{c}_{\boldsymbol{i}}^{\mathbf{(}\boldsymbol{l}\mathbf{)}}$. For the initial graph level, ${\tilde{\boldsymbol{h}}}_{\boldsymbol{i}}^{\mathbf{(0)}}\mathbf{=}{\bar{\boldsymbol{h}}}_{\boldsymbol{i}}^{\mathbf{(0)}}\mathbf{=}\boldsymbol{f}_{\boldsymbol{i}}$. The input feature for the next level’s base clustering function *φ* is then the concatenation of $\tilde{h}_{i}^{(l+1)}$ and $\bar{h}_{i}^{(l+1)}$.

## Protein System Network

The 6-layer BC protein system network (**Data S1**) generated by the MPGNN-HiLander model contained a gene column, followed by five additional columns which map to nodes in various levels of the hierarchical tree. The root level, “level 0”, is omitted from the data as only one root node exists. Downwards traversal of the tree is followed by an increase in level number (i.e. Level 0 → Level 1 → … → Level N). Each layer contains clusters of protein systems indicated by a cluster number from zero (0) ascending. HOST system IDs were numbered in ascending order of the six levels, continuing the numbering of the previous level.

## GNN Performance Metrics

Pairwise F-score (Fp) was used to measure predictive performance using precision and recall of the results. Fp is calculated using the following equations, where $T$ represents truth cluster, $P$ represents predicted cluster, and $pairs(\ldots)$ represents instance pairs generated from the same cluster.

Equation S1

$$pR=\frac{|pairs\left( P \right)\cap pairs\left( T \right)|}{|pairs\left( T \right)|}$$

Equation S2

$$pP=\frac{|pairs\left( P \right)\cap pairs\left( T \right)|}{|pairs\left( P \right)|}$$

Equation S3

$$Fp=\frac{2\times pR\times pP}{pR+pP}$$

B-Cubed F-score (Fb) is another variant of F-score to measure predictive performance using precision and recall of the results. Fb is calculated using the following equations, where $t$ represents a specific instance in the truth cluster, $T(t)$ represents a truth cluster with same instance $t$, and $P(t)$ represents a predicted cluster with same instance $t$.

Equation S4

$$bR=\frac{1}{N}\sum_{t\in T} \frac{|P(t)\cap T(t)|}{|T\left( t \right)|}$$

Equation S5

$$bP=\frac{1}{N}\sum_{t\in T} \frac{|P(t)\cap T(t)|}{|P\left( t \right)|}$$

Equation S6

$$Fb=\frac{2\times bR\times bP}{bR+bP}$$

Normalized Mutual Information (NMI) was used to measure dependence between clusters of information. NMI is calculated as shown below, where $Y$ represents class labels, C represents cluster labels, $H\left( \ldots\right)$ represents entropy, and $I(Y;C)$ represents mutual information between Y and C.

Equation S7

$$NMI(Y,C)=\frac{2\times I(Y;C)}{[H\left( Y \right)+H\left( C \right)]}$$

## Cytoscape Network Setup

The hierarchical node output of the MPGNN-HiLander model was transformed into a network input containing parent node (source node, number), child node (target node, number), genes (target attribute, string[]), and size (target attribute, number). Significance (target attribute, string) was added to the node table to represent hotspots as a combination of survival/mutation burden (**Table S2 and S3**), DepMap signal (**Table S4**), and CMap signal (**Table S5**).

# Supplementary Figures


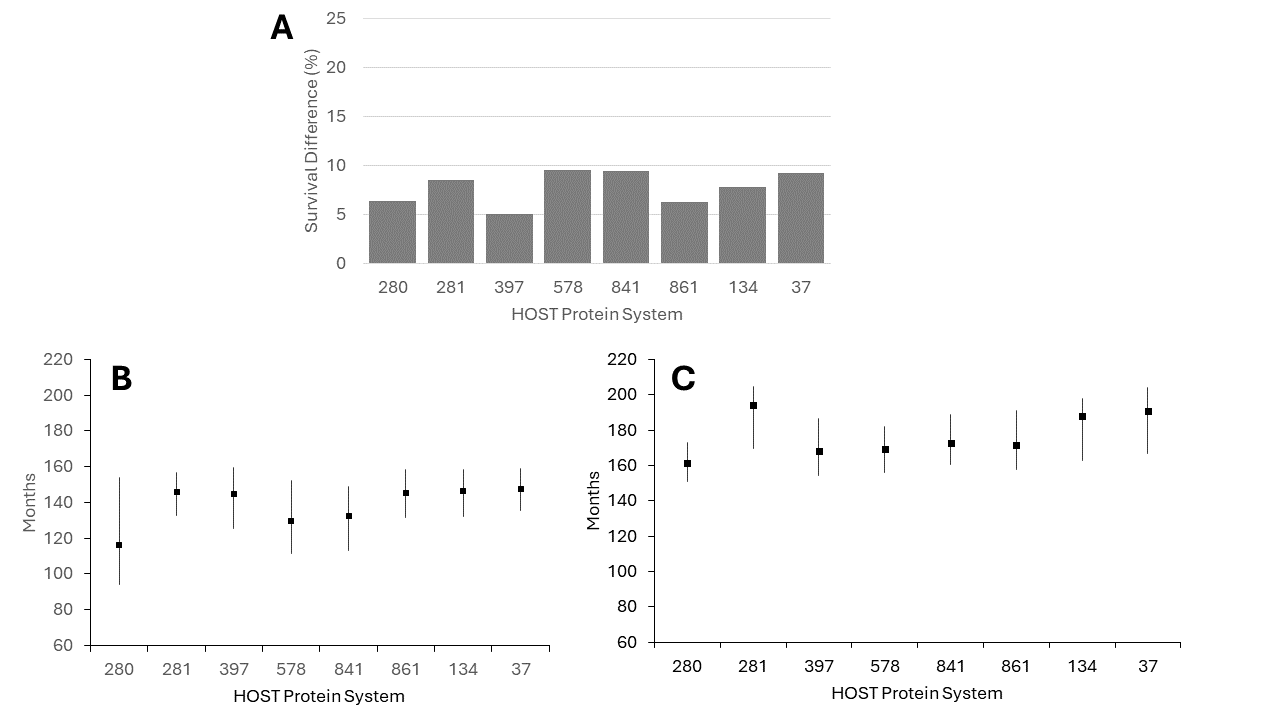


Figure S1. Comparison of significant HOST proteins systems with altered vs unaltered genes. Hotspot protein systems were selected to be significant for survival and mutation pressure, with a logrank test q-value < 0.05. Observations were taken after 10 years of prognostic testing. (A) Survival difference between altered and unaltered protein systems. (B) Survival rate of protein systems with altered genes. (C) Survival rate of protein systems with unaltered genes.

# Supplementary Tables

**Table S1. Summary of key metrics for the 10 ESM models.** M stands for million, B for billion, and performance refers to the precision of protein contact prediction.

**Table S2. HOST systems under significant survival pressure.** Survival hotspots were identified using a group-lasso algorithm on gene-level mutation data from Zheng *et al.*’s paper [1] and HOST protein systems. Significance is determined with a threshold of q-value < 0.05.

**Table S3. HOST systems under significant mutation pressure.** Mutation hotspots were identified using a group-lasso algorithm on gene-level meta-survival effects from Smith and Sheltzer’s paper [2] and HOST protein systems. Significance is determined with a threshold of q-value < 0.05.

**Table S4. HOST systems with cellular dependencies of BC cell lines.** Cellular dependencies were identified using a group-lasso algorithm on DepMap dependency data [3] and HOST protein systems. Significance is determined with a threshold of q-value < 0.05.

**Table S5. Chemical perturbagens targeting HOST systems.** Chemical perturbations were identified using a group-lasso algorithm on CMap perturbation data [4] and HOST protein systems. Significance is determined with a threshold of q-value < 0.05.

# Supplementary Data

**Data S1. Relationship data for the 6-layer BC protein system network.** Clusters are generated from the MPGNN model using data from Zheng *et al*. [1]

**Data S2. Unpruned hierarchical protein system tree (HOST).** The unpruned HOST tree contains all nodes imported from **Data S1**.

**Data S3. Pruned hierarchical protein system tree (HOST).** The pruned HOST tree contains all significant nodes from **Data S2**. Insignificant nodes were pruned using a bottom-up breadth first search approach to produce **Figure 2**.

# Supplementary References

[1] Zheng F, Kelly MR, Ramms DJ, Heintschel ML, Tao K, Tutuncuoglu B, Lee JJ, Ono K, Foussard H, Chen M, et al. Interpretation of cancer mutations using a multiscale map of protein systems. Science. 2021;374(6563):eabf3067. doi:10.1126/science.abf3067

[2] Smith JC, Sheltzer JM. Genome-wide identification and analysis of prognostic features in human cancers. Cell Reports. 2022;38(13):110569. doi:10.1016/j.celrep.2022.110569

[3] Tsherniak A, Vazquez F, Montgomery PG, Weir BA, Kryukov G, Cowley GS, Gill S, Harrington WF, Pantel S, Krill-Burger JM, et al. Defining a Cancer Dependency Map. Cell. 2017;170(3):564-576.e16. doi:10.1016/j.cell.2017.06.010

[4] Subramanian A, Narayan R, Corsello SM, Peck DD, Natoli TE, Lu X, Gould J, Davis JF, Tubelli AA, Asiedu JK, et al. A Next Generation Connectivity Map: L1000 Platform and the First 1,000,000 Profiles. Cell. 2017;171(6):1437-1452.e17. doi:10.1016/j.cell.2017.10.049
